# Effects of Team-Based Learning on Students’ Teamwork, Learning Attitude, and Health Care Competence for Older People in the Community to Achieve SDG-3

**DOI:** 10.3390/ijerph19116632

**Published:** 2022-05-29

**Authors:** Shang-Yu Yang, Cheng Liu, Pei-Lun Hsieh

**Affiliations:** 1 Department of Healthcare Administration, College of Medical and Health Science, Asia University, Taichung 41354, Taiwan; henry879019@yahoo.com.tw (S.-Y.Y.); 108211012@live.asia.edu.tw (C.L.); 2 Department of Nursing, College of Health, National Taichung University of Science and Technology, Taichung 40401, Taiwan

**Keywords:** teamwork/team-based learning, community care, community health assessment, nursing competencies, sustainable development goals (SDGs)

## Abstract

Background: Team-based learning (TBL) was studied in several preclinical settings, but evidence for its effectiveness in community nursing education is scant. A community health care nursing course was developed, and nursing students engaged in TBL to achieve Sustainable Development Goal 3. Purpose: This study aimed to examine the effect of TBL model integration on students’ learning attitude, community understanding, and community care competence for achieving SDG 3 and determine the extent to which the TBL model altered students’ nursing competence for providing community health care. We compared the effect of TBL and traditional learning (TL) in terms of community health care knowledge objectives. Methods: TBL was employed as the teaching strategy to guide students’ discussion of community care issues, allowing them to fully utilize the knowledge acquired in their community practice. We used an unblinded crossover design, and 99 students participated in the community health nursing course. Results: The results demonstrated that TBL improved participants’ community understanding and enhanced their skills for assessing and fulfilling community needs. The experimental and control groups differed significantly in their TBL performance, learning attitude, and nursing competencies. The performance of those who engaged in TBL was higher than that of those who engaged in TL on all community issues. TBL appears to be a more effective method than TL in terms of achieving nursing students’ knowledge objectives. Conclusions: Regarding practical application, the proposed intervention enables nursing students to acquire professional knowledge related to community aging health care and nursing skills, and establish partnerships with community residents. This facilitates the achievement of the United Nations’ sustainable development goal of ensuring healthy living and promoting well-being at all ages.

## 1. Introduction/Background

Sustainable Development Goal 3 (SDG 3), which relates to good health and well-being, is among the 17 SDGs established by the United Nations in 2015 [1]. In terms of official wording, the goal is to “ensure healthy lives and promote well-being for all at all ages”. Population aging is a major global trend that affects all countries. According to the SDG’s 2030 Agenda, it is important to ensure that people of all ages and social classes can achieve these goals, and that the most vulnerable groups, which include the elderly, are paid special attention to in an attempt to solve the elders’ problems of exclusion, vulnerability, and intersectional discrimination, so that the elderly are able to successfully age within the community. This can be accomplished by implementing many cross-disciplinary and health promotion activities in the community to achieve the sustainable development goals [1,2].

Nurses provide professional health care services to individuals as well as their families and communities and are therefore considered protectors of community health. Accordingly, their work corresponds to SDG 3 of the United Nations [2,3]. Through data collection and community health assessment, nurses identify the needs of community residents and determine the priority of community care issues, thereby pursuing the goal of comprehensive community health care. Community health assessments help nurses understand the characteristics of a community and determine its cultural differences, abilities, preoccupations, and motivations for problem solving. By working together with the community, nurses identify factors that are beneficial or harmful to community health, formulate community health care plans, and collaborate with other professionals and community workers to improve community health, enhancing the quality of health-related activities required in the community [3,4,5].

It was emphasized that encouraging health care professionals to view themselves as members of a global village is of great importance in a community. In order to respond to the increasing value attached to global and community health in the face of changes, and to nurture a competent and capable health care workforce, educational institutions should make adjustments in their curricula [6]. Nurses are mainly responsible for carrying out national public health policies, particularly in terms of health care and disease prevention [4]. Health promotion and illness prevention, the provision of health education to community residents, and the promotion of behavioral changes to enable people to take control of their health are all included in the community health care competencies [4,5,6].

A team-based learning (TBL) model involves a personal or group learning process with immediate feedback. Through active learning and mutual support among group members, learning goals are achieved collaboratively through group discussion of knowledge acquired in classroom settings [7,8]. TBL differs from conventional teacher-centered and student-centered instruction. In this approach, a learner reaches a consensus with team members through group discussion and accumulates basic knowledge during the learning process. Questions are proposed and discussed, thereby helping teams complete collaborative tasks. In line with recent advances in network technology, teaching strategies based on TBL models were modified to reduce the time spent creating printed tests and learning materials for students. Assigning students to groups provides them with more time for self-reflection and problem solving. This enables students to take the initiative in learning; ensures the adequate implementation of self-learning and group learning–based assessment; improves immediate feedback and interaction between teachers and students; and facilitates self-learning and team collaboration for task completion [7,8,9,10].

TBL involves three phases, namely the preparation phase, the experiment phase, and the results sharing and feedback phase [11,12]. In the preparation phase, students are divided into teams and reading materials are assigned before the class for the students to become familiar with the course content. The experiment phase involves the completion of the individual readiness assurance test and group readiness assurance test. The third phase is results sharing and feedback. During the phase of core course concept application, scenarios that involve probing questions are implemented. In this phase, all teams work on the same problems and are asked to explain how they arrived at their solution. Through activities ensuring frequent feedback, out-of-class preparation, and in-class collaboration with peers, students can enhance their interpersonal communication skills, engagement, comprehension, and critical thinking [7,12,13].

Most studies on TBL have examined improvements in end-of-course grades, test performance, knowledge, and the classroom engagement of practicing professionals in medicine and other disciplines [14]. However, the effects of TBL on the problem-solving ability, nursing knowledge, and practical performance of nursing students were only explored in a few studies. Therefore, the present study examines the effects of TBL on critical thinking, knowledge, and care competencies in relation to SDG 3 in community settings [3].

## 2. Research Question

What is the effect of TBL model–based interventions on students’ learning attitude and community health care competencies in achieving SDG 3 ?

## 3. Research Motivation

With the proportion of community-dwelling older adults exceeding 16% of the total population in 2021, Taiwan has become an aging society as defined by the United Nations. In response to population aging, older community members must retain a favorable health status that allows for independent living and aging in place. This study was based on the framework of the community-as-partner model [15,16]. The study objective was to see how students could apply the concepts they acquired in TBL to health care in the community so as to encourage students to engage in spontaneous care and improve the health of community members. Through the combination of university social responsibility (USR)-related services as well as conventional classroom teaching integrated with community action services, students were encouraged to apply their acquired knowledge on community health care and nursing in practical settings and perform health assessments and services for community residents. Accordingly, health care services specific to local communities can be implemented through collaboration with community members, thereby contributing to society and assisting with the pursuance of SDG 3.

## 4. Research Purpose

A compulsory community health care and nursing course was implemented for the fourth-year nursing students of a 5-year junior college (equivalent to freshman students in regular universities) to improve their community health care and nursing knowledge. Furthermore, the course guided them to provide community health services, assess and identify community health issues, devise care-based service plans, and interact with community members. These students were encouraged to observe, understand, and reflect on the importance of community care. The goals of this study were as follows:

(1) Examine the effect of TBL model integration on students’ learning attitude, community understanding, and community care competence for achieving SDG 3.
(2) Determine the extent to which the TBL model altered students’ nursing competence for providing community health care.

## 5. Methods

### 5.1. Design and Sample

An experimental research design was used to assign the study population comprising students recruited from the nursing department of University in Taiwan into a TBL group and traditional learning (TL) group. The study period was from September 2020 to January 2021, during which the TBL group learned about SDG issues related to older people by participating in 180 min TBL sessions once per week for 6 consecutive weeks. By contrast, the TL group participated in 180 min traditional teaching sessions once per week for 6 consecutive weeks. The participants of the two groups completed a pretest and a posttest. The test items assessed their TBL, critical thinking, and community health care assessment skills. The TBL and TL groups received instruction from different teachers; pretests and posttests were conducted by the same research assistant.

A quasi-experimental study was designed to evaluate the effects of TBL on nursing students. Eligible participants were fourth-year vocational nursing students recruited according to the following inclusion criteria: no experience of TBL and no current physical or psychiatric symptoms that would impair the ability to provide informed consent or participate in this study. Ninety-nine nursing students agreed to participate; they were enrolled in the trial and were randomly allocated to one of the two groups. The trial had no dropouts. Data from 50 students in the experimental (TBL) group and 49 students in the control group were analyzed.

**Figure 1:**
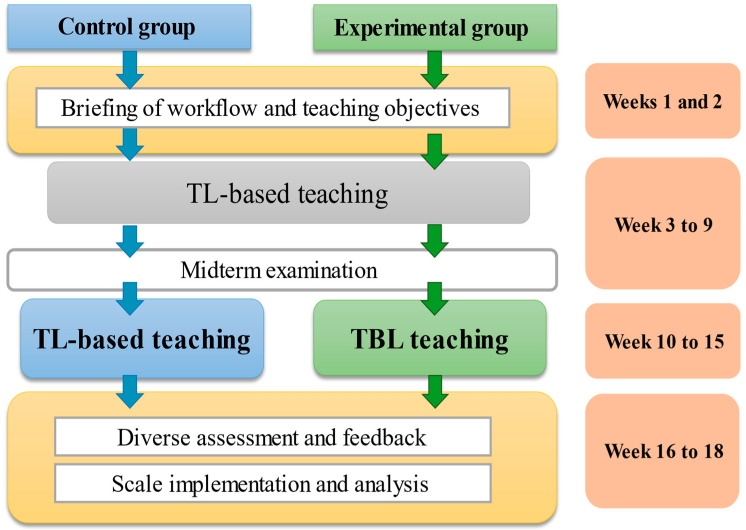
The research design process.

The participants were senior nursing students taking a community health nursing course who voluntarily agreed to participate after the study purpose, method, and expected effects were explained to them. To estimate the sample size of this study, the power threshold was set to 0.8, and the α value for significance was set to 0.05 (two tailed). Therefore, the required effect size for the two groups was 80%, and each group needed to consist of 26 participants. The participants were requested to sign an informed consent form before the intervention began. One week prior to the intervention, a pretest was conducted to collect data on demographics, TBL, learning attitude, critical thinking, and community health care competence. The experimental group received the TBL intervention for 6 weeks, and the control group received general classroom instruction for 6 weeks. Within 1 week following the completion of teaching intervention, a posttest was conducted for both groups, and the test content was identical to that of the pretest. Figure 1 presents the research design of this study.

### 5.2. Instruments

The instruments used in this study were a community understanding scale, TBL scale, and health care competence scale. The TBL scale was revised after references [17,18]; it covers collaborative tendency, communication, and problem solving. The learning attitude scale includes four dimensions, namely learning attitude, self-efficacy, team efficacy, and collaborative learning. The nursing competence scale was employed to understand the community health care competencies of nursing students; it was developed after reference to the community care nursing competence scale (15 items) for nurses [6], which includes dimensions such as community understanding and community assessment skills. The scale items were scored on a 5-point Likert scale.

Three experts in community care, older adult care, and long-term care were invited to conduct expert validity analysis by reviewing the relevance of each scale item. The experts also proposed suggestions related to wording. Accordingly, the formal scales were completed. The Cronbach’s α of the scales was 0.90 in the pilot study, 0.92 in the pretest, and 0.95 in the posttest, indicating favorable internal consistency.

### 5.3. Data Collection and Procedures

#### Research Flow

**Figure 2:**
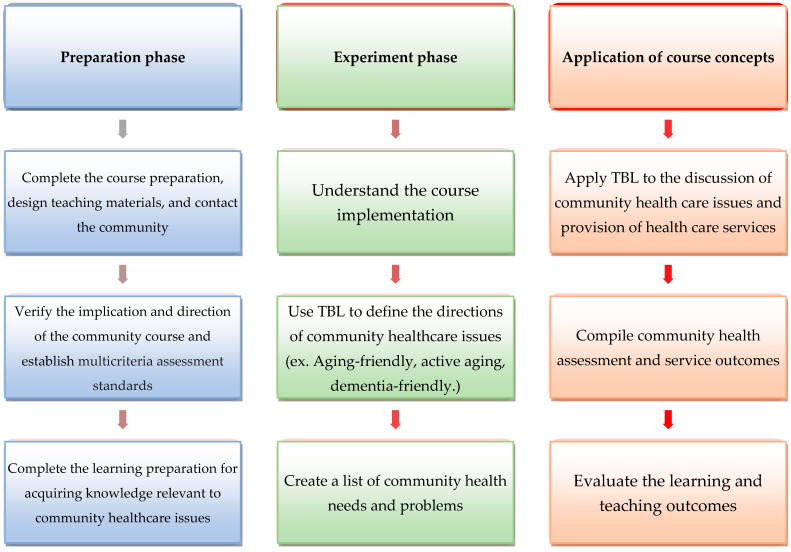
TBL phases.

In accordance with course planning, the research procedures comprised three phases, namely the preparation phase, the experiment phase, and the results sharing and feedback phase (as presented in Figure 2).

The first phase (the preparation phase; from week 1 to 9): The students participated in classroom learning to acquire knowledge related to community aging health care issues. This equipped them with prerequisite knowledge for subsequent learning activities.

The second phase (the experiment phase; from week 10 to 15): The students visited a community neighboring the older people campus to identify community aging health issues through the community-as-partner model.

The third phase (the results-sharing and feedback phase; from week 16 to 18): While participating in TBL, the students provided community health services. Subsequently, the students showcased their achievement through presentations, and learning and teaching outcome evaluations were performed.

### 5.4. Data Analysis

The collected data were analyzed using SPSS 25.0 (IBM, Inc., Armonk, NY, USA). Descriptive statistics were calculated, including numbers and percentages for the demographic characteristics of both groups and means and standard deviations (SDs) for TBL-related factors and health care competencies, including collaborative tendency, communicative tendency, problem-solving tendency, team efficacy, collaborative learning, community understanding, and community assessment skills. Homogeneity tests were conducted before the intervention; a chi-squared (Fisher’s exact probability) test, *t*-test, and analysis of covariance (ANCOVA) test were conducted for determining homogeneity between the TBL factors and health care competencies of both groups. Differences between the two groups after the intervention were determined using an independent *t*-test. Changes in health care competencies before and after the intervention in each group were determined using a paired *t*-test.

### 5.5. Ethical Approval

Ethical approval for human research was obtained from the Central Regional Research Ethics Committee of China Medical University, Taichung, Taiwan (reference No. CRREC-109-132; approved on 25 August 2020). Mandarin Chinese versions of the questionnaire and consent form were filled out by the participants. To ensure adherence to research ethics standards, the researcher explained the study purpose and research process to the participants and informed them about the protection of their personal rights.

## 6. Results

### 6.1. Differences in the Demographic Characteristics and Test Results between the Two Groups

The mean age of the participants was 18.57 (SD = 0.51) years for the experimental group and 18.79 (SD = 0.98) years for the control group, with no significant difference in age between the groups. Most of the participants in both groups were female. Furthermore, both groups were relatively homogeneous in terms of personal relationship status, degree of score with their nursing major, academic performance, and preferred learning method. The results of Fisher’s exact test revealed nonsignificant differences in the demographic variables of the experimental and control groups. (Table 1).

### 6.2. Community Care Practice with the Pursuance of SDG 3

After the intervention, the students’ understanding of the community was graded with scores ranging from 1 to 5. The mean pretest score of the experimental group was 2.62, and the mean posttest score was 4.04—a score increase of 1.42. The mean pretest score of the control group was 2.69, and the mean posttest score was 3.04, with a score increase of 0.35. The score of the experimental group was higher than that of the control group. However, the analysis of covariance (ANCOVA) results reveal a nonsignificant between-group difference (*t* = 11.59, F = 0.005, and *p* = 0.944), indicating that both groups improved their understanding of the community after the course, and the extent of improvement was similar between the two groups.

Regarding the skills for community needs assessment, the mean pretest score of the experimental group was 3.28 ± 0.57, and the mean posttest score was 3.96 ± 0.57, with an increase of 0.68. The mean pretest score of the control group was 2.94 ± 0.69, and the mean posttest score was 3.33 ± 0.69, with an increase of 0.4. The ANCOVA results reveal a significant between-group difference (*t* = 4.99, F = 8.37, *p* = 0.005), demonstrating that on-site community practice enhanced the students’ competence for the community needs assessment.

An independent samples t-test was conducted to determine between-group differences in the pretest community understanding scores. The *t*-value of the variable was −0.768, and a nonsignificant difference was noted. For between-group differences in pretest community assessment scores, the *t*-value was 2.681, and a significant difference was noted. The mean score of the experimental group was higher than that of the control group. For between-group differences in posttest community understanding scores, the *t*-value was 11.591, and a significant difference was observed. The mean score of the experimental group was higher than that of the control group. For between-group differences in posttest community assessment scores, the *t*-value was 4.980, and a significant difference was noted. The mean score of the experimental group was higher than that of the control group. A paired sample *t*-test was employed to determine the pretest–posttest differences in the two variables related to community practice, namely community understanding and community assessment, in the experimental group. According to the analysis results presented in the table, the *t*-values for community understanding and community assessment were −15.646 and −6.263, respectively, and the *p*-values of the two variables were less than the significance threshold (0.05), indicating a significant difference in community understanding and evaluation between the pretest and posttest in the experimental group.

### 6.3. TBL Model-Based Learning Outcomes

Regarding TBL, the mean score of the experimental group was 4.50 (SD = 0.52). The highest scores were found for the communication dimension (mean = 4.56 ± 0.48), followed by team efficacy (mean = 4.51 ± 0.54) and collaborative learning (mean = 4.49 ± 0.57). The mean score of the control group was 4.24 (SD = 0.60). The highest scores were also found for the communication dimension (mean = 4.33 ± 0.64), followed by team efficacy (mean = 4.29 ± 0.58), and collaborative tendency (mean = 4.24 ± 0.53). Overall, the scores of the experimental group exceeded those of the control group, verifying that the proposed intervention can enhance students’ team collaboration.

An independent samples t-test was conducted to understand between-group differences in the five team collaboration variables, namely collaborative tendency, communicative tendency, problem-solving tendency, team efficacy, and collaborative learning; the *t*-values for the aforementioned five variables were 1.994, 1.790, 2.616, 2.092, and 2.569, respectively. Nonsignificant differences were observed between the two groups.

The mean score of the experimental group for learning attitude was 4.56 (±0.39), and that of the control group was 4.50 (±0.46). The mean scores of the experimental and control groups for self-efficacy were 4.21 (±0.36) and 4.21(±0.65), respectively. The mean critical thinking scores of the experimental and control groups were 4.35 (±0.40) and 4.29 (±0.45), respectively; the score of the experimental group was higher than that of the control group. Of the critical thinking items, the score was the highest for the item “I listen carefully to others’ statements during discussion” (mean = 4.52 ± 0.50), followed by “I try to apply new insights or concepts” (mean = 4.50 ± 0.51) and “before making a decision, I try to predict the possible outcomes of all alternatives” (mean = 4.46 ± 0.50). This indicated that the students in the experimental group tended to listen to the thoughts of other people and were willing to learn and apply new concepts. An independent samples t-test was conducted to determine between-group differences in the learning attitude, self-efficacy, community understanding, and community assessment variables of the learning attitude dimension; the *t*-values for these variables were 0.652, 0.008, 11.59, 1.733, and 4.98, respectively, and a significant between-group difference was observed in community understanding and community assessment (Table 2).

## 7. Discussion

TBL is an effective teaching methodology for achieving learning outcomes in undergraduate nursing students and exploring the generic competencies, academic performance, and skills developed by the students [7]. Therefore, nurses should exercise caution when visiting clients in the community and should obtain up-to-date knowledge of emerging infectious disease and prevention measures. Furthermore, nurses should instruct the public on disease prevention and provide referrals to competent professionals to control hazardous community health care scenarios [4]. After TBL and traditional lecture-based classes were held, health care competencies, including collaborative tendency, communicative tendency, problem-solving tendency, team efficacy, collaborative learning, level of community understanding, and community assessment skills, were evaluated.

No significant differences were noted between the two groups at pretest in terms of demographic characteristics and core competencies. However, we noted improvements in both groups in all health care competencies after the intervention, with greater improvements achieved by the experimental group. A comparison of the posttest results of the two groups revealed that the experimental group obtained significantly higher scores than the control group did in community health assessment skills, communication, problem solving, and self-efficacy. The participants in the TBL group had the highest scores in communication, indicating that nurses should be equipped with strong communication competencies because these enable them to establish nurse–patient relationships and understand clients’ concerns when devising and revising care plans; this finding accords with results reported in the literature [5,7,19]. The results of this study demonstrate that communication competencies affect nursing students’ perception of empowerment; this finding is in line with those in the literature [4].

The findings related to nursing students’ community health care competencies are consistent with previous studies [12,13]. None of the participants experienced clinical situations directly, but the experimental (TBL) group participants potentially received better training to manage community issues because of their repeated learning and immediate feedback as they progressed through the three stages of preparation, readiness assurance, and course concept application.

The results in Table 2 indicate that after both groups of students took part in the course, the arrangement of community visits enhanced the community assessment skills of the experimental group students and enabled them to gain a further understanding of community issues. This demonstrates the necessity of arranging community-based courses in nursing education [14,16]. Relevant studies have reported that compared with TL groups, TBL groups obtained higher overall scores in communication, team efficacy, and collaborative tendency [7,20,21]; these findings are consistent with those in this study. The proposed intervention can effectively improve students’ TBL performance and motivate them to achieve SDG 3 in community settings. In regard to learning attitude, the score of the TBL group was higher than that of the TL group, indicating that TBL encouraged students to learn and apply new concepts by listening to others’ ideas.

Most students participating in TBL prepared outside of class prior to the in-class sessions; thus, they were prepared to actively engage in the class and encourage other students to achieve better results. They fostered the sharing of various viewpoints and ideas and were prepared to apply their knowledge in community settings [22,23]. Teamwork encourages knowledge sharing to solve problems in clinical scenarios. Active team discussions enhance decision making, and through feedback from their teammates, students can develop problem-solving skills and gain opportunities for self-reflection on their role in a team [7,14,24].

## 8. Limitations

Several limitations should be considered when interpreting the results of this study. First, the scales for the TBL and TL groups were self-administered. Although these scales were widely used in previous research, and their adequate reliability and validity were demonstrated, they cannot fully reflect actual team collaboration and nursing competencies. Second, the participants were all recruited from central Taiwan, and the number of participants was limited, which constrained the generalizability of the study results. Third, this study did not involve a randomized controlled experiment. Although nonsignificant between-group differences were observed in demographic variables, bias could still exist in the study. The outcomes of TBL-based and TL-based interventions for nursing students pursuing SDG 3 should therefore be further examined. Despite the aforementioned limitations, this study provided valuable data on the comparisons of the two instructional interventions regarding their outcomes for improving the community health care and nursing competencies of nursing students. The study results can provide a reference for community health care professionals and caregivers to select effective, empirical service intervention plans for community settings.

## 9. Conclusions

The study results indicate that following the 6-week intervention, the participants of the TBL group made substantial progress in team collaboration, learning attitude, and community health care competencies. A further comparison between the two groups in terms of TBL performance indicated that the TBL group achieved significantly higher scores in community understanding and assessment competencies. This study adds to the growing body of evidence suggesting the effectiveness of the TBL method in the achievement of knowledge objectives for nursing professional students in community settings. The scope for amending university policies and associated curricula will be considerable, and in nursing education, university curricula are required to comply with accreditation when making more use of the TBL-based model in educational courses.

## Figures and Tables

**Table 1:** Homogeneity test related to personal attributes (*N* = 99).

Variables	Experimental Group (*n* = 50)		Control Group (*n* = 49)			
	*n*	%	*n*	%	*t*/*χ*^2^	*p*
Age (mean and SD)	18.57	0.51	18.79	0.98	0.35	0.73
Sex						
Male	4	8	5	10.20	1.33	0.49
Female	16	92	44	89.80		
Level of Score with nursing major					0.11	0.95
High	19	38.00	21	42.86		
Medium	27	54.00	25	51.02		
Low	4	8.00	3	6.12		
Average scores for previous academic achievements					0.22	0.97
>90	5	10.00	6	12.24		
80–89	26	52.00	24	48.98		
70–79	17	34.00	18	36.73		
<70	2	4.00	1	2.04		

**Table 2:** Between-group differences in mean posttest scores for TBL outcomes, learning attitude, and nursing abilities (*N* = 99).

Scale/Dimension		Group	Number of Persons	Mean	Standard Deviation	F	*p*-Value	*t*	*p*-Value
TBL	Collaborative tendency	EG	50	4.436	0.511	0.091	0.764	1.994	0.049
		CG	49	4.229	0.524				
	Communicative tendency	EG	50	4.555	0.485	2.474	0.119	1.790	0.077
		CG	49	4.355	0.622				
	Problem-solving tendency	EG	50	4.488	0.516	1.575	0.212	2.616	0.010
		CG	49	4.184	0.636				
Learning attitude	Team efficacy	EG	50	4.510	0.542	0.290	0.592	2.092	0.039
		CG	49	4.276	0.573				
	Collaborative learning	EG	50	4.491	0.572	0.114	0.736	2.569	0.012
		CG	49	4.190	0.598				
	Learning attitude	EG	50	4.560	0.387	8.491 **	0.004	0.652	0.516
		CG	49	4.504	0.458				
	Individual self-efficacy	EG	50	4.210	0.365	18.071 ***	0.000	0.008	0.994
		CG	49	4.209	0.646				
Nursing abilities	Community understanding	EG	50	4.040	0.402	0.005	0.944	11.59 ***	0.000
		CG	49	3.041	0.455				
	Community assessment	EG	50	3.960	0.570	8.365 **	0.005	4.98 ***	0.000
		CG	49	3.327	0.689				

Note: ** *p* < 0.01; *** *p* < 0.001. EG: experimental group. CG: control group.

## Data Availability

The data presented in this study are available on request from the corresponding author.
